# Resting-state connectivity within the brain’s reward system predicts weight loss and correlates with leptin

**DOI:** 10.1093/braincomms/fcab005

**Published:** 2021-02-02

**Authors:** Liane Schmidt, Evelyn Medawar, Judith Aron-Wisnewsky, Laurent Genser, Christine Poitou, Karine Clément, Hilke Plassmann

**Affiliations:** 1 Control-Interoception-Attention Team, Institut du Cerveau et de la Moelle épinière (ICM), Inserm UMR 1127, CNRS UMR 7225, Sorbonne Université, 75013 Paris, France; 2 Laboratoire de Neuroscience Cognitive, Ecole Normale Supérieure, Inserm U960, 75005 Paris, France; 3 Sorbonne Université, Inserm, UMRS Nutrition et Obésités; Systemic Approaches (NutriOmics), 75013 Paris, France; 4 Nutrition Department, CRNH Ile de France, Pitié-Salpêtrière Hospital, Assistance Publique Hôpitaux de Paris, 75013 Paris, France; 5 Visceral Surgery Department, Assistance Publique Hôpitaux de Paris, Pitié-Salpêtrière Hospital, 75013 Paris, France; 6 Marketing Area, INSEAD 77305, Fontainebleau, France

**Keywords:** brain valuation system, leptin, obesity, resting-state connectivity, weight change

## Abstract

Weight gain is often associated with the pleasure of eating food rich in calories. This idea is based on the findings that people with obesity showed increased neural activity in the reward and motivation systems of the brain in response to food cues. Such correlations, however, overlook the possibility that obesity may be associated with a metabolic state that impacts the functioning of reward and motivation systems, which in turn could be linked to reactivity to food and eating behaviour and weight gain. In a study involving 44 female participants [14 patients with obesity, aged 20–63 years (mean: 42, SEM: 3.2 years), and 30 matched lean controls, aged 22–60 years (mean: 37, SEM: 1.8 years)], we investigated how ventromedial prefrontal cortex seed-to-voxel resting-state connectivity distinguished between lean and obese participants at baseline. We used the results of this first step of our analyses to examine whether changes in ventromedial prefrontal cortex resting-state connectivity over 8 months could formally predict weight gain or loss. It is important to note that participants with obesity underwent bariatric surgery at the beginning of our investigation period. We found that ventromedial prefrontal cortex–ventral striatum resting-state connectivity and ventromedial–dorsolateral prefrontal cortex resting-state connectivity were sensitive to obesity at baseline. However, only the ventromedial prefrontal cortex–ventral striatum resting-state connectivity predicted weight changes over time using cross-validation, out-of-sample prediction analysis. Such an out-of-sample prediction analysis uses the data of all participants of a training set to predict the actually observed data in one independent participant in the hold-out validation sample and is then repeated for all participants. In seeking to explain the reason why ventromedial pre-frontal cortex–ventral striatum resting-state connectivity as the central hub of the brain’s reward and motivational system may predict weight change over time, we linked weight loss surgery-induced changes in ventromedial prefrontal cortex–ventral striatum resting-state connectivity to surgery-induced changes in homeostatic hormone regulation. More specifically, we focussed on changes in fasting state systemic leptin, a homeostatic hormone signalling satiety, and inhibiting reward-related dopamine signalling. We found that the surgery-induced increase in ventromedial prefrontal cortex–ventral striatum resting-state connectivity was correlated with a decrease in fasting-state systemic leptin. These findings establish the first link between individual differences in brain connectivity in reward circuits in a more tonic state at rest, weight change over time and homeostatic hormone regulation.

## Teaser

Schmidt *et al.* report that connectivity at rest within the brain’s reward system predicted changes in weight, differentiated between lean and participants with obesity, increased after gastric bypass surgery and correlated to circulating levels of the satiety hormone leptin. These findings show neural and hormonal determinants of weight change.

## Graphical Abstract

**Graphical Abstract:**
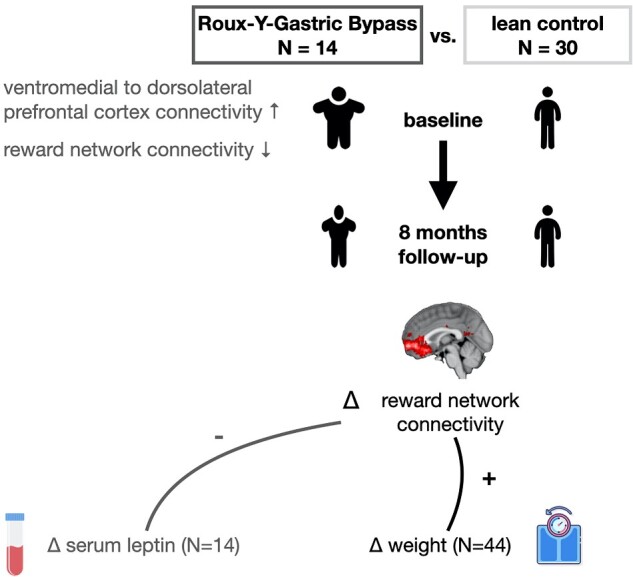

## Introduction

Despite the high prevalence of obesity worldwide, its neurobiological underpinnings and whether they can be used to formally predict weight change remain poorly understood. Research in cognitive neuroscience tends to focus on differences in task-based activity between obese and lean participants using functional magnetic resonance imaging (fMRI), finding that exposure to high-calorie foods alters activity in brain regions involved in reward and motivation processing (Rothemund *et al.*, 2007; Stice *et al.*, 2008; Volkow *et al.*, 2008; Stoeckel *et al.*, 2009; Scholtz *et al.*, 2014), taste processing (Dagher, 2012; Scharmuller *et al.*, 2012) and cognitive control (Brooks *et al.*, 2013; Pursey *et al.*, 2014).

Another stream of research has investigated tonic differences in the brain activity of participants with obesity and lean participants by capturing resting-state connectivity (RSC) among large-scale brain networks. Resting-state fMRI measures brain activity at rest, when participants are instructed to do nothing in the MRI scanner. This measure was first discovered in the late 90s when Biswal *et al.* (1997) observed that activity between brain regions of the sensorimotor system was fluctuating synchronously, suggesting an intrinsic functional organization that was active in the absence of any explicit task. This finding has since been replicated for many other brain regions, providing meaningful insights into the intrinsic anatomical and functional organization of the brain and how disease can alter this organization. These studies investigating differences in RSC between lean participants and those with obesity found differences in the salience, reward, default mode, prefrontal and temporal lobe networks (Kullmann *et al.*, 2012; Coveleskie *et al.*, 2015; García-García *et al*., 2015; Wijngaarden *et al.*, 2015; Doornweerd *et al.*, 2017). RSC in these brain systems and others was shown to be altered by bariatric surgery-based weight-loss interventions (Frank *et al.*, 2014; Olivo *et al.*, 2017; Wiemerslage *et al.*, 2017; Li *et al.*, 2018). It is important to note that all of these studies have not applied a formal, out-of-sample predictive analysis, but are using simple correlation, regression or analysis of variance within sample analysis.

Taken together, the above-cited studies show the importance of the pre-frontal brain circuits involved in decision making and reward processing, yet their role in predicting out-of-sample weight status [body mass index (BMI), weight, etc.] has never been tested. Work most closely related to ours, looking at formal predictions of BMI using resting-state fMRI [in combination with Diffusion-Tensor-Imaging (DTI), Park *et al.*, 2015] and taking a whole-brain approach, found most prominently the ventromedial prefrontal cortex (vmPFC) structure-to-function connectivity together with other regions to predict BMI. However, the specific role of vmPFC RSC in predicting weight loss has never been investigated.

Investigating the specific role of vmPFC RSC is an important contribution to the literature because prior studies using fMRI have suggested that the vmPFC is a key region of the brain’s reward-related valuation system that encodes both expected and experienced value (Bartra *et al.*, 2013). More specifically related to food decision making, the vmPFC is involved in dietary decision making (Plassmann *et al.*, 2007, 2010) and self-control (Hare *et al.*, 2009, 2011; Hutcherson *et al.*, 2012). Individual differences in vmPFC anatomy are a marker of dietary regulatory success during dietary self-control (Schmidt *et al.*, 2018). However, no research, to date, has tested whether RSC with the vmPFC as seed is sensitive to obesity and can predict weight change, which was the *first goal* of this study.

Roux-en-Y gastric bypass (RYGB) offers a unique and effective theoretical model for the question of this study because it reveals whether the relationship between the RSC with vmPFC as seed is merely correlated with obesity or whether it also changes as a function of it. In other words, it allows us to investigate whether people who gained weight and became obese exhibit higher RSC in the vmPFC than people with normal corpulence, or whether weight change induced through RYGB surgery might alter such RSC. The latter finding would suggest that obesity is *not* merely the result of a stable pre-disposition to being more responsive to rewards at rest captured by RSC in reward-processing regions such as the vmPFC (as often assumed), but may also be explained by an alternative hypothesis, i.e., that obesity—and the metabolic profile linked to it—is related to reward sensitivity captured by vmPFC RSC, which could contribute to explaining the reason *why* vmPFC RSC might predict weight loss in our study.

Thus, the *second goal* of our article was to shed first light on a potential role of the homeostatic hormone leptin to drive the changes in vmPFC RSC. To explore one potential mechanism, we focussed our investigations on leptin because it directly regulates hunger and food intake via both homeostatic and reward-related pathways: a high leptin level in the blood signals to the hypothalamus to stop food intake and the accumulation of energy through the white adipose tissue cells that secrete leptin into the blood. Leptin is therefore necessary to maintain energy accumulation at equilibrium (Montague *et al.*, 1997; Dhillon *et al.*, 2006). In addition, leptin has been shown to inhibit dopamine receptors in the nucleus accumbens, suggesting it plays an inhibitory so-called hedonic aspects of hunger and food intake (for a review, see Palmiter, 2007). In addition to leptin’s role in hormonal food intake regulation via the inhibition of reward signalling, previous research has also shown that before RYGB surgery most individuals with obesity have very high levels of leptin when in a fasted state, yet its ability to signal satiety is impaired (Myers *et al.*, 2010). After RYGB surgery, the level of leptin drops rapidly and its ability to signal satiety improves (Faraj *et al.*, 2003). Thus, a second contribution of this article is to explore whether the extent to which RYGB surgery reduces vmPFC RSC is correlated with individual differences in surgery-induced changes in fasting-state serum leptin.

## Materials and methods

### Ethical considerations and open science statement

This project is formed from amendments to two pre-existing clinical trials, Microbaria (NCT01454232) and Leaky Gut (NCT02292121), both of which received ethical approval from Assistance Publique–Hôpitaux de Paris (AP-HP). We amended the AP-HP ethical protocols to include a separate brain imaging session with the patients before and on average 8 months after the RYGB surgery, where leptin was again sampled, given our hypotheses on the potential involvement of leptin. Bariatric surgery approval was acquired during multidisciplinary meeting and after an optimal surgery preparation as required by French and international recommendations. For the brain scanning of the lean control participants, ethical approval was given by the Institut National de la Santé et de la Recherche Médicale (INSERM). The study was conducted in accordance with the Declaration of Helsinki. Participants gave informed consent. Code and anonymized data sets analysed in the study are available from the corresponding authors on request, in accordance with European GDPR regulations.

### Experimental set-up

The resting-state data presented in this article were acquired as part of a multi-study project including different experimental tasks such as task-based fMRI and analysis of blood and faecal samples for metabolic and gut microbiome profiling. The results of those measures are presented elsewhere (e.g. Aron-Wisnewsky *et al.*, 2019); the focus of this article is whether changes in RSC before and after bariatric surgery can predict changes in weight over time. The scanning session consisted of a brief introduction and training, two task-based fMRI sessions, a structural fMRI scan, and the resting-state fMRI scan presented in this article.

Data were collected at two time points (T0 and T8) separated by at least 6 months. The participants with severe obesity underwent RYGB surgery on average 17 weeks (±4 weeks) after the T0 fMRI scans. The post-surgery assessments were conducted on average 8 months (±0.6 months) after RYGB surgery. For the lean participants, assessments were separated on average by 8 months (±0.3 months).

Patients with obesity were followed in the department of nutrition at the Specialized Obesity Centre for Obesity and Obesity Surgery at Pitié-Salpêtrière Hospital in Paris. fMRI data were collected at the Centre for Neuroimaging (CENIR) at the Paris Brain Institute (ICM) at Pitié-Salpêtrière Hospital in Paris. The lean participants’ brains were also scanned twice at the same facilities of the CENIR to capture changes over time.

#### Participants

A total of 64 female participants were enrolled at T0, including 45 lean participants and 19 with severe obesity. We recruited only female participants in an attempt to keep gender influences constant (Rolls *et al.*, 1991). Additional standard fMRI inclusion criteria were right-handedness, normal to corrected-to-normal vision, no history of substance abuse or any neurological or psychiatric disorder and no medication or metallic devices that could interfere with performance of fMRI. The lean controls and participants with obesity were recruited based on their BMI, which was on average 22 ± 0.3 kg/m^2^ for the lean participants and 45 ± 1 kg/m^2^ for the candidates for bariatric surgery, in accordance with international guidelines (for more details on clinical characteristics and body composition, see Table 1). Lean participants were recruited by public advertisement in the Paris area. Participants with obesity were recruited by two co-authors of the study who were care providers of the patients following them through their pre- and post-surgery treatment course.

**Table 1:** Participant characteristics

Group	Age (s.e.m.) (years)	Education (s.e.m.) (years)	Weight (s.e.m.) (kg)	BMI (s.e.m.) (kg/m)	Body fat (s.e.m.) (%)	Body fat (s.e.m.) (kg)	Leptin (s.e.m.) (ng/ml)	Leptin/body fat (s.e.m.) (ng/ml/kg)	glycaemia (s.e.m.) (mmol/l)	Insulin (s.e.m.) (mUl/l)
Lean *N* = 40	*37 (2), n.s.*	6.5 (0.2)	62 (1)	22 (0.3)	27 (1)	17 (1)	9 (1)	0.5 (1)	4 (0.1)	4 (1)
obese T0 *N* = 16	*42 (3), n.s.*	5 (0.4)	119 (3)	45 (1)	51 (1)	62 (2.5)	70 (7)	1 (0.1)	6 (0.4)	28 (5)
obese T8 *N* = 14			85 (4)	34 (1)	45 (1)	42 (2)	25 (3)	1 (1)	5 (2)	10 (1)

Mean participant socio-demographic, bodily and systemic characteristics with standard error of the mean in brackets: Patients differed significantly in all measures, *except age*, before (T0) and after (T8) surgery and compared to lean participants, respectively (*P < *0.05, two-sampled, two-tailed *t*-test). Education is described as years of education after high school. Glycaemia was measured with chemiluminescent technology (Cobas^®^, Roche, Switzerland). Serum insulin was measured with immunoassay technology (LiaisonXL^®^, Diasorin, France). Serum leptin was determined using radioimmunoassay kits (Linco Research, St. Louis, MO, USA).

Of the 64 individuals recruited for the study, 20 participants were excluded before starting our analyses due to the following *pre-defined* exclusion criteria: two lean and three participants with obesity were excluded because of extensive head motion (≥3.5 mm), 10 lean participants were excluded because they did not return for their 8-month follow-up fMRI evaluation and three lean and two participants with obesity had incomplete rsfMRI data. Hence, a total of 44 (30 lean and 14 obese) participants were included in all analyses concerning within-participant time effects (i.e. T0 versus T8) and group by time interactions. [Note: We were able to perform analyses concerning between-participant group effects (i.e. obese versus lean) at baseline (T0) for 56 participants (40 lean, 16 obese) who had available data at T0.]

Participants were in a fasted state during the blood-sampling session to extract baseline fasting-state parameters, but not in a fasted state during the brain scanning session. Perceived hunger, measured before the brain scanning session, did not vary between groups (*β*_group_ = 0.48, se = 0.95, *P *=* *0.61) or time points (*β*_time_ = 0.5, se = 0.8, *P *=* *0.5), nor was there a significant interaction group by time point (*β*_group × time_ = −0.6, se = 0.6, *P *=* *0.3).

#### Roux-en-Y gastric bypass surgery

RYGB surgery is reserved for the most severe forms of obesity (BMI, ≥40 kg/m^2^ or BMI ≥35 kg/m^2^ with obesity-related comorbidities) (Fried *et al.*, 2014). The surgeon creates a small gastric pouch directly linked to the distal small intestine with a gastro-jejunal anastomosis. The remaining part of the stomach and the proximal small intestine are bypassed, creating a Y-Roux limb (for details, see Aron-Wisnewsky *et al.*, 2012). Ingested food thus goes directly from the newly created gastric pouch to the rest of the small intestine, which reduces the nutrients and calories absorbed from food.

In our study, the RYGB surgery was performed laparoscopically. All participants were clinically assessed before and at 1, 3 and on average 8 months post-surgery, as recommended by international guidelines (Fried *et al.*, 2014). The clinical assessments included obesity-related diseases and anthropometric measures estimated by whole-body fan beam dual-energy X-ray absorptiometry (Hologic Discovery W, software v12.6, 2; Hologic, Bedford, MA, USA), as detailed in Ciangura *et al.* (2010). Variables included weight, BMI and total body fat in kilogram and percentage (Table 1). Patients were also clinically assessed based on the following criteria: depression, using the Beck Depression Inventory; alcohol abuse, using the Alcohol Use Disorders Identification Test; nicotine consumption, using the Fagerstrom test; dietary restraint, using the Three-Factor Eating Questionnaire and diabetes (clinical assessment). Glycaemia was assessed by measuring blood glucose levels before (fasting condition) and after a standardized meal test. (Test results are shown in Supplementary Table 1.) These clinical assessments were not done for the lean participants.

#### Blood hormone sampling

Blood samples were collected once from the lean participants (at T0), and twice for participants with obesity, before (T0) and 8 months after RYGB (T8). Venous blood samples were collected in the fasting state (12-h fasting) for the determination of glycaemia, insulinemia and leptin. Glycaemia was measured with chemiluminescent technology (Cobas^®^, Roche, Switzerland). Serum insulin was measured with immunoassay technology (LiaisonXL^®^, Diasorin, France). Serum leptin was determined using radioimmunoassay kits (Linco Research, St. Louis, MO, USA). The Leaky Gut and Microbaria clinical trials also included the measurement of GLP1; unfortunately, for the patients included in this study, these measurements were not reliably done due to technical issues.

### Brain imaging data

#### Image acquisition

Resting-state fMRI scanning was conducted during a 10-min scanning sequence after participants took part in several task-based fMRI sessions. Resting-state activation was assessed after task-evoked brain activation for all participants and at all time points, which was crucial for group and time point comparisons. Implementing this design allowed us to keep influences of task-evoked brain activation on resting-state brain activity constant across these comparisons. Moreover, for data-quality reasons, total scanning time (including task-based and resting-state fMRI) was limited to an hour to reduce the discomfort of obese participants that has been linked to the fMRI environment, which is an important factor to consider when combining behavioural testing with fMRI.

Participants were instructed to keep their eyes closed and relax, but not to fall asleep.

T2*-weighted echo planar images with blood–oxygen-level-dependent contrast were acquired using a 3T Siemens Verio scanner. An eight-channel phased array coil was used to assess whole-brain resting-state activity with the following ascending interleaved sequence: Each volume comprised 40 axial slices, TR = 2 s, TE = 24 ms, 3-mm slice thickness; 0.3-mm inter-slice gap corresponding to 10% of the voxel size; field of view = 204 mm; flip angle = 78°. For each participant, a total of 304 volumes were obtained. The first five volumes of the resting-state scan session were discarded to allow for T1 equilibrium effects. A single high-resolution T1-weighted structural image (magnetization-prepared rapid gradient-echo) was acquired, co-registered with the mean echo planar image, segmented and normalized to a standard T1 template. Normalized T1 structural scans were averaged across lean and obese participants, respectively, to allow group-level anatomical localization.

#### Preprocessing

Data were analysed using Statistical Parametric Mapping software (SPM12; Wellcome Department of Imaging Neuroscience) along with the Functional Connectivity toolbox (CONN toolbox: www.nitrc.org/projects/conn, RRID: SCR_009550). Pre-processing in SPM included spatial realignment to estimate head motion parameters. This preprocessing step was done prior to slice-time correction, because slice-time correction can lead to systematic under-estimates of motion when it is performed as a first pre-processing step (Drysdale *et al.*, 2017). After re-alignment, pre-processing included the standard steps: slice-time correction, co-registration, normalization using the same transformation as structural images, spatial smoothing using a Gaussian kernel with full width at half maximum of 8 mm and temporal band pass filtering between 0.01 and 0.1 Hz.

#### Nuisance signal removal

Nuisance signal removal was performed on the pre-processed time-series data with the CONNv16 toolbox; it included linear and quadratic de-trending to adjust for scanner drift, to remove nuisance signals related to head motion and to physiological variables by means of regression analyses. More specifically, the nuisance regression included 18 head motion parameters calculated during spatial re-alignment (roll, pitch, yaw and translation in three dimensions, plus their first and second derivatives), non-neuronal signals from eroded white matter and cerebrospinal fluid masks, and regressors for outlier volumes. Individual white-matter and cerebrospinal fluid masks were obtained by segmentation of each participant’s structural magnetization-prepared rapid gradient-echo image into tissue probability maps using SPM12. The white-matter and cerebrospinal fluid masks were further eroded to reduce partial volume effects. We used CONNv16’s ART-based function to identify outlier volumes with a global signal *Z*-value threshold of 3 and an inframe displacement threshold of ≥0.5 mm, corresponding to the most conservative setting in the CONNv16 toolbox (95th percentile in normative sample). This more conservative setting is important in light of recent first findings that head movement might be linked to weight loss (Beyer *et al.*, 2020). It should be noted that the nuisance signal regression and band-pass filtering were performed simultaneously, only on volumes that survived head motion censoring. We used a rather lenient head motion threshold of ≥3.5 mm in order to not exclude too many of our participants with obesity, who moved significantly more than lean participants. After pre-processing, the smoothed residual time-series data, co-registered to Montreal-Neurological Institute (MNI) space, were used for the subsequent statistical analysis steps.

### Statistical analyses

We first focussed on a theory-driven, seed-to-voxel connectivity analysis with the vmPFC as a seed, and compared how vmPFC-to-voxel RSC differed between lean and obese participants at baseline. We then extracted the RSC patterns from regions that displayed a difference between groups at baseline to predict weight change over time in the main analyses.

#### Seed region of interest: vmPFC

We leveraged Neurosynth’s meta-analysis tool (Yarkoni *et al.*, 2011) and created an ROI map for the term ‘vmPFC’. Specifically, the seed ROI was defined by the neurosynth website using the ‘reverse inference’ map for ‘vmPFC’. The mask was thresholded at *P *<* *0.0001 uncorrected, after smoothing the *Z-*map with a 6-mm FWHM kernel and averaging *Z-*scores across the left and right hemispheres to create a symmetrical map. We further re-sliced each mask to the lean controls’ and obese patients’ normalized mean echo planar images to make sure that all voxels were within the vmPFC in our participant sample.

#### Determining RSC networks of interest

In order to choose the regions exhibiting RSC that would predict out-of-sample weight loss, in the first step of our analyses we looked for regions that showed a significant difference in RSC between lean and obese participants at T0 by means of a multiple regression analysis. The latter correlated the averaged blood–oxygen-level-dependent signal from the vmPFC seed ROI to the blood–oxygen-level-dependent signal in each voxel of the brain for each participant. The Pearson’s *r* for each voxel was then transformed into a *Z*-score using Fisher *r-*to-*z* transformations to obtain normally distributed functional connectivity coefficient maps. Individual functional connectivity coefficient maps were subjected to second-level random-effects factorial analysis of variance (2* *×* *2 ANOVA) crossing participant group (obese versus lean participants) and time point (T0 versus T8). We considered a false-discovery rate (FDR)-corrected significance threshold of *P*_FDR_ < 0.05 at the cluster level and further explored results at an uncorrected voxel-wise threshold of *P < *0.001 to report the full extent of the effects (Poldrack *et al.*, 2008). The full results of the ANOVA are summarized in Table 2 for the sake of completeness. Planned contrasts were examined between groups at baseline (T0) using cluster-corrected *P*_FDR_ < 0.05 significance thresholds.

**Table 2:** Main effect of group on vmPFC resting-state connectivity

Region	BA	Size	*x*	*y*	*z*	Peak *Z*-score
Obese > lean participants at T0						
Cerebellum		729	−20	−80	−44	5.30
		848	14	−78	−44	4.95
dlPFC	47	166	−34	10	46	4.26
Obese < lean participants at T0						
Ventral striatum		172	−10	10	−4	4.51
Hippocampus		283	22	−48	8	4.44
Obese > lean participants at T8						
IFG	45/46/47	243	−54	38	12	4.74
vlPFC	47/11	191	44	42	−12	4.15
	10/11	228	30	60	0	4.08

The peak co-ordinates and *Z*-score values are listed for lean participants compared to obese patients before at T0 and on average 8* *months after bariatric surgery at T8. All peaks surpassed a voxel-wise threshold of *P*_FDR_ < 0.05 FDR corrected on the cluster level. The *xyz* coordinates correspond to the Montreal Neurological Institute (MNI) space.

Abbreviations: dlPFC, dorsolateral prefrontal cortex; IFG, inferior frontal gyrus; vlPFC, ventrolateral prefrontal cortex.

Against this background, we used RSC between the vmPFC-vStr and vmPFC–dorsolateral pre-frontal cortex (dlPFC) for our main analysis since these were the only ROIs that satisfied our criterion determining sensitivity to obesity.

#### Main analysis: out-of-sample prediction of weight change over time by vmPFC-to-voxel RSC

To test whether weight change could be predicted from vmPFC-to-voxel RSC, we conducted the following leave-one-participant-out predictive analysis. First, *Z*-values of vmPFC-to-voxel RSC were extracted for each participant and averaged across the voxels from the two ROIs that displayed a significant difference between obese and lean participants at baseline: the vStr (MNI = [−10, 10, −4]) and the dlPFC (MNI = [−34, 10, 46]). The average *Z*-values for each ROI were then, respectively, used to conduct 44 linear regressions that determined independent weights of the vmPFC-to-voxel connectivity on weight loss (kilogram at T8 minus kilogram at T0) for more than 43 participants (leaving out the 44th participant) following equation (1):

(1)  T8kg -T0kg =β0+ βROI× ZvmPFC-ROI +ϵ

Each time, the weight (βROI) of the vmPFC-ROI connectivity on weight loss obtained from 43 participants together with the *Z*-value for vmPFC-ROI connectivity extracted from the ROI in the left-out participant was regressed to predict weight loss for the left-out participant $\hat{y(}l\mathrm{eft}\;\mathrm{out}\;\mathrm{participant}$) using the *glmval* function in matlab as in equation (2):

(2) $$\hat{y}(\mathrm{left}\;\mathrm{out}\;\mathrm{participant})=\beta \mathrm{ROI}\left(43\right)\times \;\mathrm{ZvmPFC}-\mathrm{ROI}(\mathrm{left}\;\mathrm{out}\;\mathrm{participant})\;+\epsilon$$

Last, we quantified the association between the predicted and the observed levels of weight loss by using Pearson’s correlation, which was tested for significance by using both parametric one-sampled *t*-tests and non-parametric permutation tests (10* *000 permutations).

#### Hormone correlation analysis

We conducted correlation analyses to explore whether the changes due to the weight loss intervention in vmPFC-vStr RSC co-varied with the changes in leptin per kilogram body fat lost after surgery as a hormonal marker of homeostatic control of food intake. To this aim, Pearson’s correlation coefficient *ρ* was calculated as in equation (3):

(3) $$\rho_{x,y}\;=\;\frac{\mathrm{cov}(x,y)}{\sigma_{x}\sigma_{y}}$$

with *cov* corresponding to the covariance of *x* and *y* and *σ* corresponding to the standard deviation of *x* and *y*. Specifically, *x* corresponded to the change in raw serum leptin per kilogram body fat lost after surgery according to equation (4):

(4) $$x=\frac{\left(\frac{ng}{ml}l{\mathrm{eptin}}_{T0}\;-\;\frac{ng}{ml}l{\mathrm{eptin}}_{T8}\right)}{({kg\;\mathrm{body}\;\mathrm{fat}}_{T0}\;-\;{kg\;\mathrm{body}\;\mathrm{fat}}_{T8})\;}$$

Because leptin is produced by white adipose tissue cells, the change in ng/ml leptin after surgery co-varies significantly with kilogram body fat lost after surgery (Pearson’s *ρ* = 0.68, *P *=* *0.007). We therefore considered the ratio as a measure of interest in order to account for the dependency between body fat and leptin. The ratio of the changes in leptin per kilogram body fat lost from T0 to T8 reflects the change of serum leptin levels per kilogram body fat lost after bariatric surgery. This ratio *x* was correlated to the change in vmPFC-vStr connectivity after surgery *y*. *Y* was computed by following equation (5):

(5) $$y=\;z_{T8}-z_{T0}$$

Mean connectivity values (*z_vmPFC-vStr_*) were extracted for each obese participant at T0 and T8 from the vStr cluster that displayed a significant connectivity to the vmPFC seed ROI for the group (obese > lean) by time point (T8 > T0) interaction (MNI coordinates = [−10 6 −2], *P < *0.001 uncorrected, extend threshold 50 voxels). We used the interaction ROI in the vStr to rule out non-specific changes in vmPFC-vStr RSC over time that are not related to bariatric surgery.

The significance of Pearson’s correlation coefficients was tested by conducting both parametric one-sampled *t*-tests and non-parametric permutation tests, which are less sensitive to individual outliers, and estimated the 95% confidence intervals (CI) for correlations due to chance based on 10* *000 permutations of the observed data. In more detail, the permutation shuffled 10* *000 times the observed true values in order to calculate 10* *000 Pearson’s correlation coefficients. The distribution of these correlation coefficients and hence the 95% CI revealed how much otherwise uncorrelated random variables will be correlated negatively and positively. Any observed, true positive or negative correlation should, if significant, lay outside the 95% CI for permuted chance correlations within a sample of n participants.

### Data availability

Data and software/code for analysis are available upon reasonable request from the corresponding author.

## Results

### Differences in RSC of the vmPFC in participants with obesity compared to lean participants at baseline

**Figure 1:**
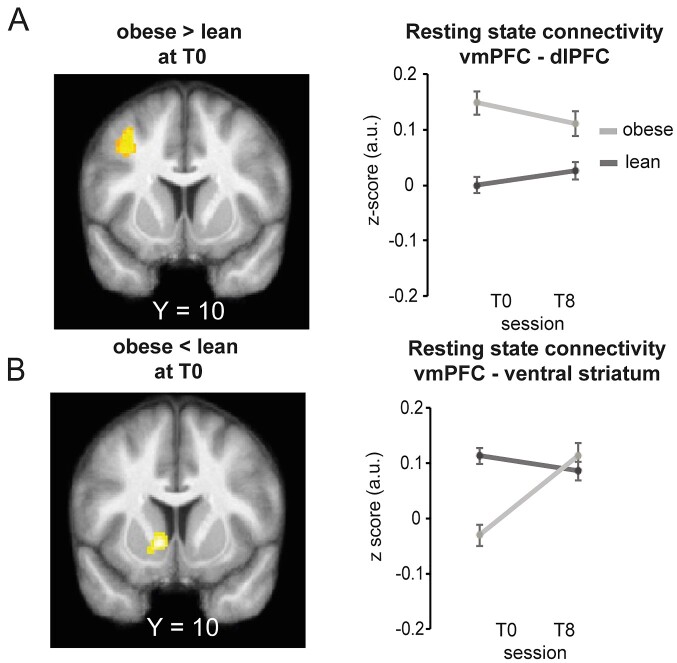
**Comparisons of vmPFC-to-brain RSC in lean participants and those with obesity before and after bariatric surgery.** SPMs of the seed-to-voxel RSC between the vmPFC seed ROI and the rest of the brain, at baseline (T0) (*n *=* *56) in **A** obese > lean participants and **B** obese < lean participants. Significant voxels are displayed for visualization purposes in orange at *P *<* *0.001 uncorrected, with an extent threshold *k *=* *166 and *k* = 172 voxels, corresponding to a FDR corrected threshold of *P*_FDR_ < 0.05 at the cluster level for each contrast, respectively. SPMs are superimposed on the average structural image obtained from the lean participants. The [*x*, *y*, *z*] co-ordinates correspond to MNI coordinates and are taken at maxima of interest. The line graphs on the right of each SPM show average correlation coefficients between RSC of the seed region, the vmPFC and the **A** dlPFC and **B** vStr at baseline (T0) in lean (dark grey) and obese (light grey) participants.

We first investigated differences in RSC in the brain’s valuation system with the vmPFC as seed between the obese and the lean participants at T0. *Post hoc* comparisons between groups revealed that at baseline (T0), severely obese patients compared to lean participants displayed stronger vmPFC connectivity to cognitive regulation nodes such as the dlPFC (cluster-corrected *P*_FDR_ < 0.05; Fig. 1a and Table 2). We also found weaker vmPFC connectivity to motivational nodes such as the vSTR (cluster-corrected *P*_FDR_ < 0.05; Fig. 1b and Table 2) at baseline (T0). Thus, we used these two regions that were sensitive to obesity at baseline as regions of interest for our out-of-sample prediction.

### Out-of-sample prediction of weight change over time across all participants

**Figure 2:**
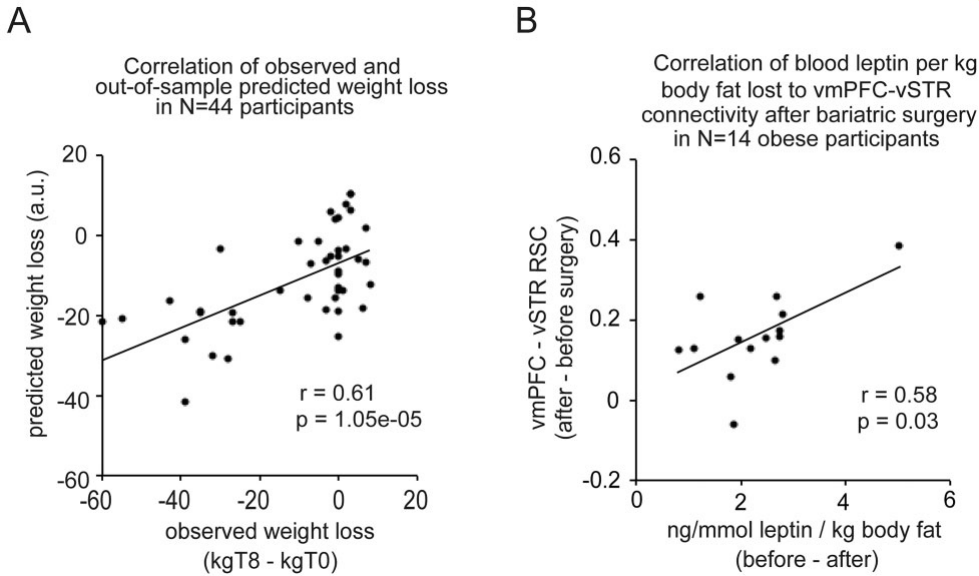
**Out-of-sample prediction of weight change, and correlations to leptin levels of vmPFC-vSTR RSC.** (**A**) Scatterplots depict in all participants (*N* = 44) the correlation between observed weight loss (kilogram body weight at T8 minus kilogram body weight at T0) and predicted weight loss obtained from an out-of-sample cross-validation of the association between weight loss and vmPFC-vStr RSC. Dots correspond to obese participants. (**B**) Scatterplots show, in obese participants (*n* = 14), the change observed after, compared to before, bariatric surgery in vmPFC-vStr RSC (average correlation coefficients) as a function of ng/mmol leptin per kilogram body fat lost. Dots correspond to obese participants.

We investigated whether changes in vmPFC-to-voxel RSC could predict changes in participants’ weight between two time points using a leave-one-sample-out predictive analysis. When we based the prediction of weight change on information about the vmPFC-vStr RSC, there was a significant positive association between the predicted and the observed weight change (*r *=* *0.61, *P *=* *1.05e* *−* *05, 95% CI due to chance: −0.24 to 0.25; Fig. 2a).

However, when using information about vmPFC-dlPFC RSC out-of-sample prediction of weight change was non-significant (Pearson’s *r *=* *0.15, *P *=* *0.3, 95% CI due to chance: −0.25 to 0.25). These findings indicate a specific role of vmPFC to vStr RSC for weight change.

### Individual differences in relative fasting-state leptin correlate with changes in vmPFC-vStr RSC

We explored how much the change in vmPFC-vStr RSC after RYGB surgery was correlated with changes in serum leptin, taking into account the reduction of body fat. Secreted by adipose tissue, the hormone leptin is known to contribute to signalling satiety and to stop food intake via inhibition dopamine receptors in the ventral tegmental area and melanocortin (i.e. MC4) receptors in the hypothalamus. As expected, leptin and body fat were elevated in participants with obesity before surgery, and decreased significantly post-surgery [% body fat: *t*(13) = 9.9, *P *<* *0.001; kilogram body fat: *t*(13) = 13.7, *P < *0.001; ng/ml leptin: *t*(13) = 5.6, *P < *0.001; two-tailed, paired *t*-test; Table 1]. Examining the link between the decrease in leptin per kilogram of body fat loss after RYGB surgery to the increase in vmPFC-vStr RSC after surgery, we found a significant positive correlation (Pearson’s *ρ* = 0.58, *P *=* *0.03, 95% CI due to chance: −0.46 to 0.46; Fig. 2b). In other words, obese participants who lost more circulating leptin per unit of fat mass post-RYGB were also those who had the most important increase in vmPFC-vStr RSC post-RYGB surgery.

## Discussion

Our study provides first evidence—using an out-of-sample prediction across all participants—that changes in RSC between the vmPFC and the vStr predict how much weight participants will lose over a period of 8 months. These are two key regions within the brain’s valuation system that are involved in processing reward and motivation (Knutson *et al.*, 2005; Rangel *et al.*, 2008). To the best of our knowledge, our study is the first to uncover an association between weight change over time and the connectivity of neural hubs at rest within the brain’s valuation and reward system.

To further quantify this somewhat reversed inference using non-task-based resting-state fMRI data, Neurosynth.org’s association test maps were leveraged. They consist of *Z*-scores in voxels activated more consistently *and* specifically in studies that mention the terms used here (e.g. ‘reward’ and ‘emotion’) than for studies that do not (Poldrack, 2010; Yarkoni *et al.*, 2011). To demonstrate the specific link with the vmPFC and vStr for value and reward, we searched Neurosynth.org’s database for the terms ‘reward’, ‘value’, ‘cognitive control’ and ‘emotion’. We examined the positive *Z*-scores for our vmPFC seed ROI’s centre MNI co-ordinates (MNI = [0, 43, −8]) and our second ROI’s peak’s coordinate in the vStr that showed sensitivity to obesity (MNI = [−10, 6, −2]). Table 3 lists the *Z*-scores for the two MNI co-ordinates of interest. They reflect how much the vmPFC and vStr, respectively, are involved in the reward, valuation, emotion and cognitive control processes. They lend support to the role of the vmPFC and vStr in reward and valuation processes, rather than in emotion or cognitive control processes that could be seen as related.

**Table 3:** Positive *Z*-scores in Neurosynth’s association maps for different mental processes

Search term	vmPFC [0, 43, −8]	Ventral striatum [−10, 6, 2]	Maximum *Z*-score
Reward	6.56	14.82	28.56
Value	4.01	5.05	11.68
Cognitive control	0	0	8.94
Emotion	0	0	18.93

*Note*: Values correspond to *Z*-scores. The maximum *Z*-score corresponds to brain voxels with the maximum association to the search term, and that provide a references for the *Z*-scores of the [x, y, z] MNI coordinates of interest located our ROIs in the vmPFC and ventral striatum.

Our findings—that vmPFC-vStr RSC was attenuated among participants with obesity (versus lean participants)—parallel others using task-based fMRI that have shown a de-sensitization of the brain’s reward circuitry in response to food rewards among obese participants (Volkow *et al.*, 2013; Carter *et al.*, 2016). It has led to the stigma and interpretation that people with obesity consume more palatable foods rich in calories because they have de-sensitized brain reward circuitry. However, our results show that the alternative idea cannot be ruled out: that being obese may be linked to a metabolic profile that partly also impacts reward sensitivity. If so, then an altered reward sensitivity as captured by vmPFC-vStr RSC would not make people pre-disposed to obesity in the first place. Moreover, should the same participants be found to differ in vmPFC-vStr RSC as a function of their weight status and BMI, it would provide evidence that obesity is linked to an altered reward system at rest. This was, indeed, what we found: vmPFC-vStr RSC increased after surgery in participants with obesity and was a predictor of the magnitude of weight change in the overall sample.

An interesting next question is, what is the mechanism underlying this? Such a bi-directional link might be related to improved functioning of dopaminergic projections from the mid-brain to regions of the brain’s hedonic valuation system. This idea is supported by studies using positron emission tomography of dopaminergic functioning in obese patients, which found dopamine D2 receptor availability to increase 6 weeks post-RYGB surgery (Steele *et al.*, 2010), reaching levels similar to those observed in non-obese controls. Although we could not directly measure dopamine in our study, we could sample fasting-state serum leptin that can be indirectly linked to dopamine, as leptin acts on hypothalamic melanocortin and basal ganglia dopamine receptors to regulate energy homeostasis—notably to reduce appetite and inhibit food intake. Fasting-state leptin levels are generally high in obese patients before surgery, suggesting they may have resistance to its anorexic action (Stice *et al.*, 2008; Crujeiras *et al.*, 2015), and rapidly decrease after bariatric surgery (to a higher extent than surgery-induced decreases in fat mass) (Faraj *et al.*, 2003). Strengthening the potential link between our findings and dopamine functioning as the underlying mechanism, we found that vmPFC-vStr RSC was positively correlated with the reduction of fasting-state serum leptin (taking into account fat-mass loss) after RYGB surgery. It might suggest that the one underlying mechanism explaining the reason why changes in vmPFC-vStr RSC predict weight could be linked to hormonal homeostatic control that targets hypothalamic and dopaminergic pathways in order to influence food-related behaviour and weight loss.

However, this association does not constitute causal evidence that bariatric surgery (through body fat loss) decreases leptin levels, which then act upon dopaminergic projections from mid-brain neurons to improve the RSC in the brain’s reward-related valuation system, or that improved dopamine functioning is a result of the improved vmPFC-vStr RSC and is independent of the observed decreased leptin levels. Our study opens up opportunities for future research to investigate the causal links between vmPFC-vStr RSC changes induced by bariatric surgery, leptin and dopamine functioning (e.g. by using positron emission tomography in combination with dopamine markers).

We further found that compared with those who were lean, participants with severe obesity displayed an enhanced RSC between the vmPFC and a set of lateral prefrontal cortex regions that are associated with the cognitive regulation of affective states, working memory and the cognitive control of goal-directed action selection (Ochsner *et al.*, 2002; Wager and Smith, 2003; Charron and Koechlin, 2010). This result is in line with the findings from fMRI studies, showing an impulse control-related activation of the lateral and dorsolateral pre-frontal cortex in patients with milder obesity (Weygandt *et al.*, 2015). However, investigating connectivity at rest in participants with severe obesity, we did not find a prominent role of vmPFC-dlPFC RSC in weight loss as reported in prior task-based fMRI studies. Possible reasons could either be driven by methodological differences between studies or differences in the extent of obesity in participants. In our study sample, we imaged participants with severe obesity who qualified for RYGB surgery and were moderately restrained in their eating behaviour (Supplemental Table 1). Other possible reasons suggested by previous study using similarly characterized participants and methods (e.g. Olivo *et al.*, 2017) could be linked to the timing of our study. Olivo *et al.*’s (2017) findings suggest that RSC changes in related control regions might occur only after a longer period of time—they found RSC changes only after 12 months (but not 1 month) post-RYGB surgery.

Our study has several limitations, one being the relatively small number of participants with obesity. Studies that rely on such patients often have a smaller sample size than studies conducted in healthy participants since the recruitment of such patients is challenging, and especially difficult in longitudinal studies such as ours (which, despite a relatively low number of patients, took 3* *years to conduct). However, even with relatively small samples, studies such as ours are important to advance the understanding of the mechanisms of weight change. Another aspect to consider is the estimated effect sizes of changes in RSC before and after bariatric surgery. Prior studies, even with a small sample size (e.g. Li *et al.*, 2018, *N* = 22; Cerit *et al.*, 2019, *N* = 14) showed that the effect of bariatric surgery on RSC is quite drastic. Given the severity of the weight loss intervention, effect sizes of β parameter estimates in ROIs vary between −0.1 pre-surgery, and 0.18 post-surgery (Li *et al.*, 2018; Cerit *et al.*, 2019). Our effect sizes (*β* = −0.05 before surgery and *β* = 0.11 after surgery for vmPFC-vStr RSC, corrected for multiple comparisons) are in line with the reported effect sizes in such a patient population. One could therefore argue that this patient population may show larger effect sizes and thus may require smaller samples as compared with the studies of healthy participants.

Another limitation of our study is that we included participants with severe obesity and lean participants, populations at the two extremes of the spectrum, for our prediction analyses. The strength of our study lies in the follow-up after a successful weight loss intervention, which was the reason why we selected such extreme BMI values. To generalize our results, we call for future studies that replicate our findings using participants who are in between these extremes—that is, those who are overweight or less severely obese or severely obese waiting for surgery.

In such replications, we would also encourage researchers to make a direct, physiological assessment of whether participants are awake during resting-state scanning, for example by also taking measurements of eye movement. In our study, we asked participants to close their eyes and relied on their self-reports that they remained awake during the resting-state scan.

Another limitation concerns the relationship of our findings to other metabolic markers of obesity that (i) influence hedonic and homeostatic hunger and (ii) are changed by bariatric surgery in obese participants. Notably, insulin has been linked to weight status, resting-state activity and reward sensitivity (Heni *et al.*, 2012; Kullmann *et al.*, 2012; Tiedemann *et al.*, 2017). We found that the patients with improved insulin sensitivity after surgery also displayed stronger vmPFC-vStr RSC. However, this correlation was non-significant, potentially because of the small sample size raised above. We call for more research in this field to verify these promising trends and also put them in perspective with respect to other clinical markers of obesity (e.g. changes in regional body fat across lean and obese participants that in our study we collected only for the participants with obesity) and how they relate to changes in vmPFC-vStr RSC.

Such future study would contribute to a more complete understanding of the biological and psychological underpinnings of weight loss and obesity.

## Conclusion

The contribution of our study is in providing first evidence that vmPFC-vStr RSC (i) formally predicts weight loss and (2) is linked to RYGB surgery-induced changes in leptin levels (i.e. a marker of the hormonal homeostatic system of food intake control). It is also one of the first to integrate hedonic and homeostatic factors in controlling food intake (Berthoud 2006).

## Supplementary Material

Supplementary material is available at *Brain Communications* online.

## Supplementary Material

fcab005_Supplementary_Data

## Abbreviations

ANOVA =: analysis of variance
AP-HP =: Assistance Publique–Hôpitaux de Paris
BMI =: body mass index
CENIR =: Centre for Neuroimaging
CI =: confidence intervals
dlPFC =: dorsolateral pre-frontal cortex
DTI =: diffusion-tensor-imaging
FDR =: false-discovery rate
fMRI =: functional magnetic resonance imaging
FWHM =: full width at half maximum
GDPR =: general data privacy regulations
ICM =: Paris Brain Institute
INSERM =: Institute National de la Santé et de la Recherche Médicale
MC =: melanocortin
MNI =: Montreal-Neurological Institute
ROI =: region of interest
rsfMRI =: resting state functional magnetic resonance imaging
RSC =: resting-state connectivity
RYGB =: Roux-en-Y gastric bypass
SPM =: Statistical Parametric Mapping
TEFQ =: Three-Factor Eating Questionnaire
vmPFC =: ventromedial prefrontal cortex
vSTR =: ventral striatum

## References

[fcab005-B1] Aron-Wisnewsky J, Doré J, Clement K. The importance of the gut microbiota after bariatric surgery. Nat Rev Gastroenterol Hepatol 2012; 9: 590–8. 22926153 10.1038/nrgastro.2012.161

[fcab005-B2] Crujeiras AB, Carreira MC, Cabia B, Andrade S, Amil M, Casanueva FF. Leptin resistance in obesity: an epigenetic landscape. Life Sci 2015; 140: 57–63. 25998029 10.1016/j.lfs.2015.05.003

[fcab005-B3] Dagher A. Functional brain imaging of appetite. [Review]. Trends Endocrinol Metab 2012; 23: 250–60. 22483361 10.1016/j.tem.2012.02.009

[fcab005-B4] Doornweerd S, van Duinkerken E, de Geus EJ, Arbab-Zadeh P, Veltman DJ, IJzerman RG. Overweight is associated with lower resting state functional connectivity in females after eliminating genetic effects: a twin study. Hum Brain Mapp 2017; 38: 1–13. 10.1002/hbm.23715 PMC6867098 28718512

[fcab005-B5] Drysdale AT, Grosenick L, Downar J, Dunlop K, Mansouri F, Meng Y, et al Resting-state connectivity biomarkers define neurophysiological subtypes of depression. Nat Med 2017; 23: 28–38. 27918562 10.1038/nm.4246 PMC5624035

[fcab005-B6] Faraj M, Havel PJ, Phélis S, Blank D, Sniderman AD, Cianflone K. Plasma acylation-stimulating protein, adiponectin, leptin, and ghrelin before and after weight loss induced by gastric bypass surgery in morbidly obese subjects. J Clin Endocr Metab 2003; 88: 1594–602. 12679444 10.1210/jc.2002-021309

[fcab005-B7] Fried M, Yumuk V, Oppert JM, Scopinaro N, Torres A, Weiner R, on behalf of International Federation for the Surgery of Obesity and Metabolic Disorders—European Chapter (IFSO-EC) and European Association for the Study of Obesity (EASO), et al Interdisciplinary European guidelines on metabolic and bariatric surgery. Obes Surg 2014; 24: 42–55. 24081459 10.1007/s11695-013-1079-8

[fcab005-B8] García-García I, Jurado MA, Garolera M, Marqués-Iturria I, Horstmann A, Segura B, et al Functional network centrality in obesity: a resting-state and task fMRI study. Psychiatry Res 2015; 233: 331–8. 26145769 10.1016/j.pscychresns.2015.05.017

[fcab005-B9] Hare TA, Malmaud J, Rangel A. Focusing attention on the health aspects of food changes value signals in vmPFC and improves dietary choice. J Neurosci 2011; 31: 11077–87. 21795556 10.1523/JNEUROSCI.6383-10.2011 PMC6623079

[fcab005-B10] Hutcherson CA, Plassmann H, Gross JJ, Rangel A. Cognitive regulation during decision making shifts behavioral control between ventromedial and dorsolateral prefrontal value systems. J Neurosci 2012; 32: 13543–54. 23015444 10.1523/JNEUROSCI.6387-11.2012 PMC3689006

[fcab005-B11] Knutson B, Taylor J, Kaufman M, Peterson R, Glover G. Distributed neural representation of expected value. J Neurosci 2005; 25: 4806–12. 15888656 10.1523/JNEUROSCI.0642-05.2005 PMC6724773

[fcab005-B12] Myers MG, Leibel RL, Seeley RJ, Schwartz MW. Obesity and leptin resistance: distinguishing cause from effect. Trends Endocrinol Metab 2010; 21: 643–51. 20846876 10.1016/j.tem.2010.08.002 PMC2967652

[fcab005-B13] Ochsner KN, Bunge SA, Gross JJ, Gabrieli JDE. Rethinking feelings: an fMRI study of the cognitive regulation of emotion. J Cogn Neurosci 2002; 14: 1211–29. 10.1162/089892902760807212 12495527

[fcab005-B14] Palmiter RD. Is dopamine a physiologically relevant mediator of feeding behavior? [Review]. Trends Neurosci 2007; 30: 35–46. 10.1016/j.tins.2007.06.004 17604133

[fcab005-B15] Plassmann H, O’Doherty JP, Rangel A. Appetitive and aversive goal values are encoded in the medial orbitofrontal cortex at the time of decision making. J Neurosci 2010; 30: 10799–808. 20702709 10.1523/JNEUROSCI.0788-10.2010 PMC6634706

[fcab005-B16] Plassmann H, O’Doherty JP, Rangel A. Orbitofrontal cortex encodes willingness to pay in everyday economic transactions. J Neurosci 2007; 27: 9984–8. 17855612 10.1523/JNEUROSCI.2131-07.2007 PMC6672655

[fcab005-B17] Poldrack RA, Fletcher PC, Henson RN, Worsley KJ, Brett M, Nichols TE. Guidelines for reporting an fMRI study. [Review]. Neuroimage 2008; 40: 409–14. 18191585 10.1016/j.neuroimage.2007.11.048 PMC2287206

[fcab005-B18] Scharmuller W, Ubel S, Ebner F, Schienle A. Appetite regulation during food cue exposure: a comparison of normal-weight and obese women. Neurosci Lett 2012; 518: 106–10. 22580204 10.1016/j.neulet.2012.04.063

[fcab005-B19] Stoeckel LE, Kim J, Weller RE, Cox JE, Cook IIE, Horwitz B. Effective connectivity of a reward network in obese women. Brain Res Bull 2009; 79: 388–95. 19467298 10.1016/j.brainresbull.2009.05.016 PMC3441054

[fcab005-B20] Tiedemann LJ, Schmid SM, Hettel J, Giesen K, Francke P, Büchel C, et al Central insulin modulates food valuation via mesolimbic pathways. Nature Commun 2017; 8: 1–10. 28719580 10.1038/ncomms16052 PMC5520049

[fcab005-B21] Volkow ND, Wang GJ, Tomasi D, Baler RD. Obesity and addiction: neurobiological overlaps. Obes Rev 2013; 14: 2–18. 23016694 10.1111/j.1467-789X.2012.01031.x PMC4827343

[fcab005-B22] Wager TD, Smith EE. Neuroimaging studies of working memory: a meta-analysis. Cogn Affect Behav Neurosci 2003; 3: 255–74. 15040547 10.3758/cabn.3.4.255

[fcab005-B23] Wiemerslage L, Zhou W, Olivo G, Stark J, Hogenkamp PS, Larsson EM, et al A resting‐state fMRI study of obese females between pre‐ and postprandial states before and after bariatric surgery. Eur J Neurosci 2017; 45: 333–41. 27718507 10.1111/ejn.13428

[fcab005-B24] Wijngaarden MA, Veer IM, Rombouts SARB, van Buchem MA, Willems van Dijk K, Pijl H, et al Obesity is marked by distinct functional connectivity in brain networks involved in food reward and salience. Behav Brain Res 2015; 287: 127–34. 25779924 10.1016/j.bbr.2015.03.016

[fcab005-B25] Yarkoni T, Poldrack R, Nichols T, Essen D, Wager T. Large-scale automated synthesis of human functional neuroimaging data. Nat Methods 2011; 8: 665–70. 21706013 10.1038/nmeth.1635 PMC3146590

[fcab005-B26] Aron-Wisnewsky J, Prifti E, Belda E, Ichou F, Kayser BD, Dao MC, et al Major microbiota dysbiosis in severe obesity: fate after bariatric surgery. Gut 2019; 68: 70–82. 29899081 10.1136/gutjnl-2018-316103 PMC7143256

[fcab005-B27] Bartra O, McGuire JT, Kable JW. The valuation system: a coordinate-based meta analysis of BOLD fMRI experiments examining neural correlates of subjective value. Neuroimage 2013; 76: 412–27. 23507394 10.1016/j.neuroimage.2013.02.063 PMC3756836

[fcab005-B28] Berthoud HR. Homeostatic and non-homeostatic pathways involved in the control of food intake and energy balance. Obesity 2006; 14: 197S–200S. 17021366 10.1038/oby.2006.308

[fcab005-B29] Beyer F, Prehn K, Wüsten KA, Villringer A, Ordemann J, Flöel A, Witte AV. Weight loss reduces head motion: revisiting a major confound in neuroimaging. Hum Brain Mapp 2020; 41: 2490–4. 32239733 10.1002/hbm.24959 PMC7267971

[fcab005-B30] Biswal BB, Van Kylen J, Hyde JS. Simultaneous assessment of flow and BOLD signals in resting-state functional connectivity maps. NMR Biomed 1997; 10: 165–70. 9430343 10.1002/(sici)1099-1492(199706/08)10:4/5<165::aid-nbm454>3.0.co;2-7

[fcab005-B31] Brooks SJ, Cedernaes J, Schiöth HB. Increased prefrontal and parahippocampal activation with reduced dorsolateral prefrontal and insular cortex activation to food images in obesity: a meta-analysis of fMRI studies. PloS One 2013; 8: e60393. 23593210 10.1371/journal.pone.0060393 PMC3622693

[fcab005-B32] Carter A, Hendrikse J, Lee N, Yücel M, Verdejo-Garcia A, Andrews ZB, et al The neurobiology of “food addiction” and its implications for obesity treatment and policy. Annu Rev Nutr 2016; 36: 105–28. 27296500 10.1146/annurev-nutr-071715-050909

[fcab005-B33] Cerit H, Davidson P, Hye T, Moondra P, Haimovici F, Sogg S, et al Resting-state brain connectivity predicts weight loss and cognitive control of eating behavior after vertical sleeve gastrectomy. Obesity 2019; 27: 1846–55. 31689011 10.1002/oby.22607 PMC6839788

[fcab005-B34] Charron S, Koechlin E. Divided representation of concurrent goals in the human frontal lobes. Science 2010; 328: 360–3. 20395509 10.1126/science.1183614

[fcab005-B35] Ciangura C, Bouillot J-L, Lloret-Linares C, Poitou C, Veyrie N, Basdevant A, et al Dynamics of change in total and regional body composition after gastric bypass in obese patients. Obesity 2010; 18: 760–5. 19834464 10.1038/oby.2009.348

[fcab005-B36] Coveleskie K, Gupta A, Kilpatrick LA, Mayer ED, Ashe-McNalley C, Stains J, et al Altered functional connectivity within the central reward network in overweight and obese women. Nutr Diabetes 2015; 5: e148. 25599560 10.1038/nutd.2014.45 PMC4314578

[fcab005-B37] Dhillon H, Zigman JM, Ye C, Lee CE, McGovern R, Tang V, et al Leptin directly activates SF1 neurons in the VMH, and this action by leptin is required for normal body weight homeostasis. Neuron 2006; 49: 191–203. 16423694 10.1016/j.neuron.2005.12.021

[fcab005-B38] Frank S, Wilms B, Veit R, Ernst B, Thurnheer M, Kullman S, et al Altered brain activity in severely obese women may recover after Roux-en-Y gastric bypass surgery. Int J Obes 2014; 38: 341–8. 10.1038/ijo.2013.60 23711773

[fcab005-B39] Hare TA, Camerer CF, Rangel A. Self-control in decision-making involves modulation of the vmPFC valuation system. Science 2009; 324: 646. 19407204 10.1126/science.1168450

[fcab005-B40] Heni M, Kullman S, Ketterer C, Guthoff M, Linder K, Wagner R, et al Nasal insulin changes peripheral insulin sensitivity simultaneously with altered activity in homeostatic and reward-related human brain regions. Diabetologia 2012; 55: 1773–82. 22434537 10.1007/s00125-012-2528-y

[fcab005-B41] Kullmann S, Heni M, Veit R, Ketterer C, Schick F, Häring H-U, et al The obese brain: association of body mass index and insulin sensitivity with resting state network functional connectivity. Hum Brain Mapp 2012; 33: 1052–61. 21520345 10.1002/hbm.21268 PMC6870244

[fcab005-B42] Li G, Ji G, Hu Y, Xu M, Jin Q, Liu L, et al Bariatric surgery in obese patients reduced resting connectivity of brain regions involved with self‐referential processing. Hum Brain Mapp 2018; 39: 4755–65. 30062852 10.1002/hbm.24320 PMC6866485

[fcab005-B43] Montague CT, Farooqi S, Whitehead JP, Soos MA, Rau H, Wareham NJ, et al Congenital leptin deficiency is associated with severe early-onset obesity in humans. Nature 1997; 387: 903–8. 9202122 10.1038/43185

[fcab005-B44] Olivo G, Zhou W, Sundbom M, Zhukovsky C, Hogenkamp P, Nikontovic L, et al Resting-state brain connectivity changes in obese women after Roux-en-Y gastric bypass surgery: a longitudinal study. Sci Rep 2017; 7: Article 6616. 10.1038/s41598-017-06663-5 PMC5529553 28747648

[fcab005-B45] Park BY, Seo J, Yi J, Park H. Structural and functional brain connectivity of people with obesity and prediction of body mass index using connectivity. PLoS One 2015; 10: e0141376. 26536135 10.1371/journal.pone.0141376 PMC4633033

[fcab005-B46] Poldrack R. Mapping mental function to brain structure: how can cognitive neuroimaging succeed? [Review]. Perspect Psychol Sci 2010; 5: 753–61. 25076977 10.1177/1745691610388777 PMC4112478

[fcab005-B47] Pursey KM, Stanwell P, Callister RJ, Brain K, Collins CE, Burrows TL. Neural responses to visual food cues according to weight status: a systematic review of functional magnetic resonance imaging studies. Front Nutr 2014; 1: 7–11. 25988110 10.3389/fnut.2014.00007 PMC4428493

[fcab005-B48] Rangel A, Camerer C, Montague PR. A framework for studying the neurobiology of value-based decision making. [Review]. Nat Rev Neurosci 2008; 9: 545–56. 18545266 10.1038/nrn2357 PMC4332708

[fcab005-B49] Rolls BJ, Fedoroff IC, Guthrie JF. Gender differences in eating behavior and body weight regulation. Health Psychol 1991; 10: 133–42. 2055211 10.1037//0278-6133.10.2.133

[fcab005-B50] Rothemund Y, Preuschhof C, Bohner G, Bauknecht H-C, Klingebiel R, Flor H, et al Differential activation of the dorsal striatum by high-calorie visual food stimuli in obese individuals. NeuroImage 2007; 37: 410–21. 17566768 10.1016/j.neuroimage.2007.05.008

[fcab005-B51] Schmidt L, Tusche A, Manoharan N, Hutcherson C, Hare T, Plassman H. Neuroanatomy of the vmPFC and dlPFC predicts individual differences in cognitive regulation during dietary self-control across regulation strategies. J Neurosci 2018; 38: 5799–806. 29866743 10.1523/JNEUROSCI.3402-17.2018 PMC6246877

[fcab005-B52] Scholtz S, Miras AD, Chhina N, Prechtl CG, Sleeth ML, Daud NM, et al Obese patients after gastric bypass surgery have lower brain-hedonic responses to food than after gastric banding. Gut 2014; 63: 891–902. 23964100 10.1136/gutjnl-2013-305008 PMC4033279

[fcab005-B53] Steele KE, Prokopowicz GP, Schweitzer MA, Magunsuon TH, Lidor AO, Kuwabawa H, et al Alterations of central dopamine receptors before and after gastric bypass surgery. Obes Surg 2010; 20: 369–74. 19902317 10.1007/s11695-009-0015-4

[fcab005-B54] Stice E, Spoor S, Bohon C, Small DM. Relation between obesity and blunted striatal response to food is moderated by TaqIA A1 allele. Science 2008; 322: 449–52. 18927395 10.1126/science.1161550 PMC2681095

[fcab005-B55] Volkow ND, Wang GJ, Fowler JS, Telang F. Overlapping neuronal circuits in addiction and obesity: evidence of systems pathology. Phil Trans R Soc B 2008; 363: 3191–200. 18640912 10.1098/rstb.2008.0107 PMC2607335

[fcab005-B56] Weygandt M, Mai K, Dommes E, Ritter K, Leupelt V, Spranger J, et al Impulse control in the dorsolateral prefrontal cortex counteracts post-diet weight regain in obesity. Neuroimage 2015; 109: 318–27. 25576647 10.1016/j.neuroimage.2014.12.073

