# Supplementary material for: Resting-state connectivity within the brain’s reward system predicts weight loss and correlates with leptin
Source: Brain Commun. 2021 Feb 2;3(1):fcab005. doi: 10.1093/braincomms/fcab005 (PMC7884604; doi:10.1093/braincomms/fcab005)
Supplement: fcab005_Supplementary_Data [file fcab005_supplementary_data.pdf]

Supplementary information

Resting-state connectivity (RSC) within the brain's reward system predicts weight loss and correlates with leptin

Liane Schmidt,[1*] Evelyn Medawar,[2#] Judith Aron-Wisnewsky,[3#] Laurent Genser,[4] Christine Poitou,[3§] Karine Clément,[3§] and Hilke Plassmann[1,5*]

1. Additional clinical assessments of morbidly obese patients before surgery......................2

2. Additional statistical analysis and results: Main effect of group in RSC of the vmPFC and effect of RYGB surgery (i.e., group x time interaction)...2

3. Additional statistical analysis and results: Residual leptin and vmPFC-vStr connectivity 3

4. Additional statistical analysis and results: Insulin sensitivity and vmPFC-vStr connectivity..3

5. Supplementary Figure 1...5

6. Supplementary Figure 2...6

7. Supplementary Table 1: Clinical assessment of patients with obesity before RYGB surgery 6

8. Supplementary Table 2: Demographic and clinical data for excluded lean participants and those with obesity 7

9. Supplementary Table 3: Differences in vmPFC RSC between groups at T8. 7

1. **Additional clinical assessments of morbidly obese patients before surgery**

Before bariatric surgery, obese participants were assessed on the following: depression, using the Beck Depression Inventory (BDI); alcohol abuse, using the Alcohol Use Disorders Identification Test (AUDIT); nicotine consumption, using the Fagerstrom test; dietary restraint, disinhibition, and hunger, using the Three-Factor Eating Questionnaire (TFEQ); and diabetes (clinical assessment). Glycemia was assessed by measuring blood glucose levels before (fasting conditions) and after a standardized meal test. The test results (Supplementary Table 1) show the following: In line with bariatric surgery recommendations, obese participants were not depressed, and on average they were not characterized by alcohol abuse (mean = 6.4, s.e.m. = 1.3; abuse cutoff score ≥7, dependence cutoff score ≥11). On average, obese participants were not nicotine dependent (mean = 2.3, s.e.m. = 1.3; cutoff score for weak dependence >4);. Average severity of dietary restraint (mean = 2, s.e.m. = 0.2), disinhibition (mean = 1.4, s.e.m. = 0.1), and hunger (mean = 1.2, s.e.m. = 0.1) was weak to moderate.

2. **Additional statistical analysis and results: Main effect of group in RSC of the vmPFC and effect of RYGB surgery (i.e., group x time interaction)**

We investigated differences in RSC in the brain's valuation system with the vmPFC as seed between the obese and lean participants. In other words, we looked at the main effect of participant group irrespective of time and found that participants with obesity presented stronger vmPFC RSC to a set of frontal brain regions including the dorsolateral prefrontal cortex (dlPFC), the ventrolateral prefrontal cortex (vlPFC), and the medial prefrontal cortex (mPFC) (cluster-corrected $p_{FDR} < 0.05$). We also tested for a main effect of time, but found no differences between T0 and T8 across the whole participant sample, even at a more lenient uncorrected threshold of p<0.001.

Post hoc comparisons between groups further revealed that after 8 months (T8), participants with obesity continued to have stronger vmPFC-to-mPFC and -vlPFC RSC (cluster-corrected $p_{FDR} < 0.05$; Supplemental Figure 1, Supplemental Table 3).

We investigated whether RYGB surgery affected the RSC of the vmPFC, and, if so, whether it would affect its RSC to other brain regions involved in reward and motivation processing and control. In more detail, we compared the difference in the RSC of the vmPFC in the participants with obesity after versus before RYGB surgery to the change over time in the RSC of the vmPFC in the lean participants (i.e., the obese group > lean group by time T8 > T0 interaction). We found stronger RSC between the vmPFC and the vStr RSC for this interaction (MNI coordinates [-10 6 -2], $p_{unc} < 0.001$, extent threshold $k = 50$ voxels; Supplemental Figure 2).

3. Additional statistical analysis and results: Residual leptin and vmPFC-vStr connectivity

As a robustness check we also calculated residual leptin values by regressing out any variance of leptin explained by kg body fat. We then correlated the difference of before minus after surgery in residual leptin values to the difference in before minus after surgery in vmPFC-vStr RSC, respectively. It revealed a significant covariance ($\rho = 0.41$, $p = 0.08$, 95% CI due to chance: -0.45–0.46; for % body fat: $\rho = 0.52$, $p = 0.05$, 95% CI due to chance: -0.45–0.46).

4. Additional statistical analysis and results: Insulin sensitivity and vmPFC-vStr connectivity

Other metabolic measures such as insulin also have also been shown to be indirectly linked to dopamine (Palmiter 2007). Hence, as a robustness check we conducted a similar analysis to test the correlation of vmPFC-vStr connectivity with insulin using the Homeostatic Model Assessment of Insulin Resistance (HOMA-IR) score that reflects insulin resistance, as per equation (vi):

(vi) HOMA-IR = (GPF * IPF)/22.5

where GPF corresponds to the fasting-state glycemia, and IPF to fasting-state insulin. HOMA–IR scores were smaller after bariatric surgery than before, indicating a decrease in insulin resistance after surgery, which was significant ($t(13)=3.3$, $p=0.005$). The magnitude of this decrease ($HOMA\text{-}IR_{T8}$ – $HOMA\text{-}IR_{T0}$) was positively correlated with the change in vmPFC-vStr RSC after surgery ($r = 0.30$, $p = 0.2$), indicating the same trend as leptin, albeit not significant.

5. Supplementary Figure 1

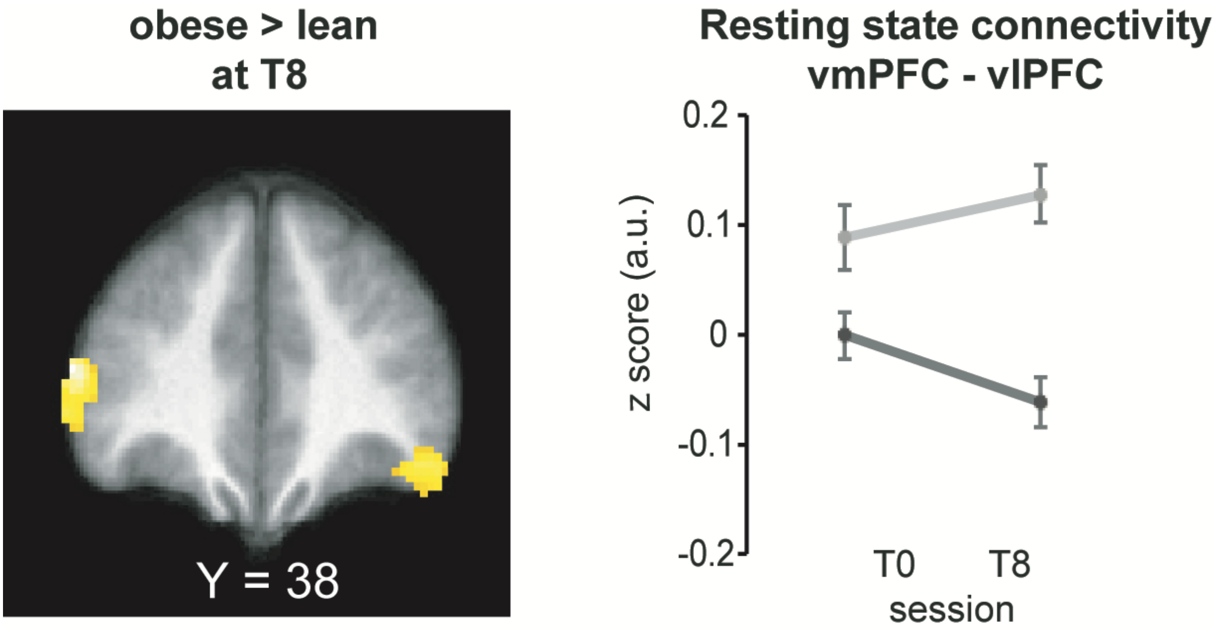

Comparisons of vmPFC to brain resting-state connectivity in lean participants compared to those with obesity after bariatric surgery (T8). Statistical parametric maps (SPMs) of the seed-to-voxel resting-the vmPFC seed ROI and the rest of the brain at 8 months post-surgery (T8) (n = 44) Significant voxels are displayed for visualization purposes in orange at p < 0.001 uncorrected, k corresponding to a false discovery rate (FDR) corrected threshold of pFDR < 0.05 on the average structural image obtained from the lean participants. The [x, y, z] coordinates correspond to MNI coordinates and are taken at maxima of interest. The line graphs on the right side depict average correlation coefficients between resting state activity of the seed region, the vmPFC, and the right vlPFC in lean (dark grey) and obese (light grey) participants.

6. Supplementary Figure 2

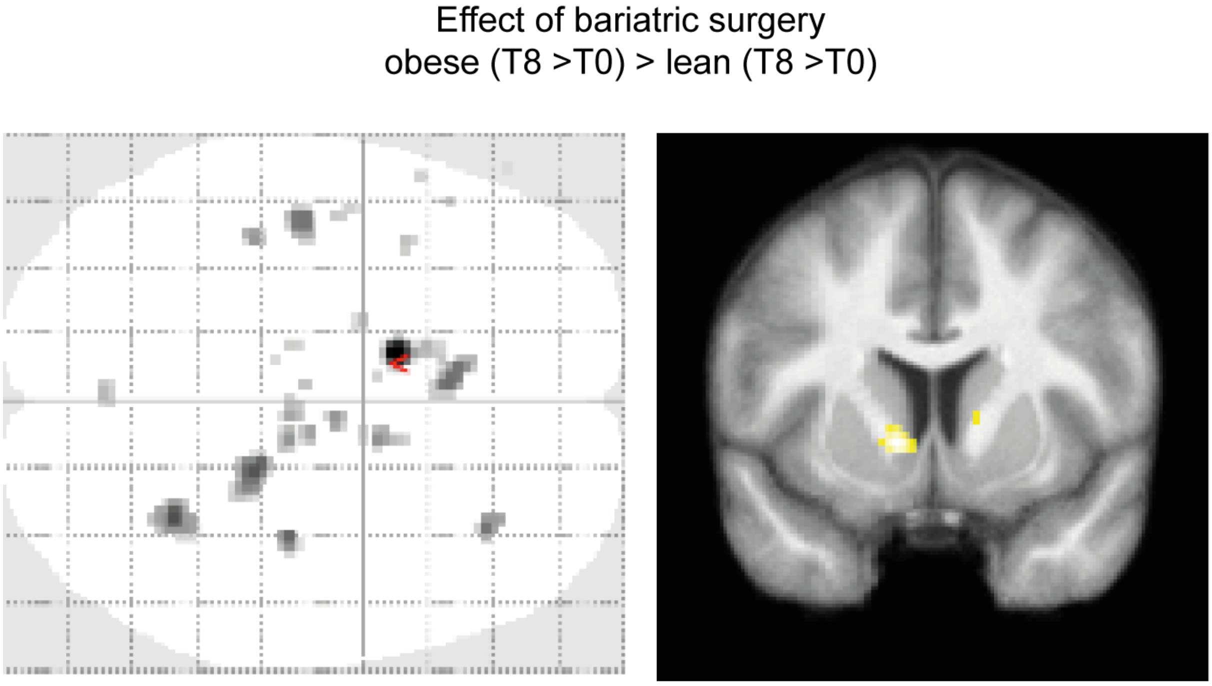

Activity in the vmPFC seed correlated significantly more to resting-state activity in the striatum in obese participants after surgery compared to before surgery and to lean participants for the time between baseline (T0) and eight months later (T8) assessments (N = 44, p < 0.001 uncorrected). on the left panel display all voxels activated on the axial slice taken at the global maximum indicated arrow. Statistical parametric maps are superimposed on the average structural image obtained from the lean participants.

7. Supplementary Table 1: Clinical assessment of patients with obesity before RYGB surgery

	Mean	s.e.m.
Beck Depression Inventory	1.3	0.4
AUDIT	6.4	1.3
Fagerstrom	2.3	1.3
Dietary restraint (TFEQ)	2.0	0.2
Dietary disinhibition (TFEQ)	1.4	0.1
Hunger (TFEQ)	1.2	0.1
% of participants with glycemia before surgery	0.8	0.1
% of participants with glycemia after surgery	0.3	0.1

AUDIT (alcohol use disorders): in women, a score of ≥7 indicates alcohol abuse, and ≥11 indicates alcohol dependence. Fagerstrom score (nicotine dependence): 0 to 2 = none, 3 to 4 = weak, 5 to 6 = moderate, 7 to 10 = strong. TFEQ score (severity of dietary restraint, disinhibition, and hunger): 1 = low, 2 = moderate, 3 = high. Glycemia reflects the number of participants with glycemia and hyperglycemia.

8. Supplementary Table 2: Demographic and clinical data for excluded lean participants and those with obesity

Group	Age (s.e.m.) years	Education (s.e.m.) years	Weight (s.e.m.) kg	BMI (s.e.m.) kg/m	Body fat (s.e.m.) %	Body fat (s.e.m.) kg	Leptin (s.e.m.) ng/ml	Glycemia (s.e.m.) mmol/l	Insulin (s.e.m.) mUI/l
Lean n = 15	38 (2)	6.5 (0.4)	64 (2)	23 (0.3)	28 (1)	17 (1)	9 (1)	5 (0.1)	45 (1)
obese T0 n = 5	41 (7)	4 (0.1)	129 (9)	47 (3.9)	53 (2.5)	68 (8)	72 (10)	6 (0.3)	31 (5)

9. Supplementary Table 3: Differences in vmPFC RSC between groups at T8.

**Obese > Lean participants at T8**						
Region	*BA*	*size*	*x*	*y*	*z*	*Peak z-score*
IFG	45/46/47	243	-54	38	12	4.74
vlPFC	47/11	191	44	42	-12	4.15
	10/11	228	30	60	0	4.08

This table reports the peak coordinates and *z*-score values for lean participants compared with those with obesity 8 months after bariatric surgery (T8). All peaks surpassed a voxel-wise threshold of $p_{FDR} < 0.05$ false discovery rate (FDR) corrected at the cluster level. The *xyz* coordinates correspond to the Montreal Neurological Institute (MNI) space. IFG: inferior frontal gyrus; vlPFC: ventrolateral prefrontal cortex.
